# Diagnostics and Decisions: Molecular Test Insights in Shared Decision-Making for Managing Respiratory Infections

**DOI:** 10.2196/81968

**Published:** 2025-11-20

**Authors:** Zachary N Goldberg, Maren Susan Fragala, Azia Evans, Steven E Goldberg

**Affiliations:** 1 Department of Otolaryngology, Health and Neck Surgery, Boston Medical Center, Boston, MA, United States; 2 Clinical and Scientific Affairs, HealthTrackRx, 1500 Interstate 35W, Denton, TX, 76207, United States, 1 (866) 287-3218

**Keywords:** shared decision-making, PCR, respiratory infections, diagnostics, antibiotic prescribing, urgent care

## Abstract

Advancements in diagnostic technologies for the evaluation of infectious disease complaints in the outpatient setting have improved the speed and accuracy of pathogen detection and created the opportunity for higher accuracy in treatment planning. The benefits of these advanced diagnostics insights can be optimized when coupled with robust shared decision-making between the patient and clinician during the clinical encounter. This manuscript describes the process for the integration of results from molecular testing for respiratory tract infection into a shared decision-making framework. It also explores how this synergy may lead to improved patient outcomes, enhanced health care delivery, and more collaborative care, while enhancing diagnosis and treatment of respiratory infections in various clinical settings.

## Introduction: Shared Decision-Making and Diagnostics

Shared decision-making (SDM) is a collaborative process in which clinicians and patients integrate clinical evidence with patient values and preferences to arrive at mutually acceptable treatment choices [1,2]. SDM was first introduced in the 1980s, with a formalized process described in 1993 that has been continuously refined over the prevailing decades [1]. The Agency for Healthcare Quality and Research offers a well-established clinician-led SDM model (ie, the SHARE approach) to promote the implementation of SDM in clinical practice; examples of SDM using the SHARE approach are shown in Table 1 [3]. Effective SDM requires transparent communication about diagnostic findings, treatment options, and potential outcomes.

**Table 1.** Clinical examples of integrating shared decision-making with next-day diagnostic tests using the SHARE [3] approach.

SHARE [3] approach	Clinical strategy	Example
Seek	Seek your patient’s participation in treatment decision-making	“ Would you be open to discussing the pros and cons of starting antibiotics now versus waiting for the test results?”
Help	Help your patient explore and compare treatment options	“We can either start antibiotics now, which may not be effective if the infection is viral, fungal, or a resistant or atypical bacteria, or wait for the test results to guide us. Starting antibiotics unnecessarily can lead to resistance and other complications. What are your thoughts on these options?”
Assess	Assess your patient’s values and preferences	“I want to understand what’s most important to you—avoiding unnecessary medications, minimizing long-term risks like antibiotic resistance, or quick relief. How do you feel about waiting until tomorrow morning for the test results?”
Reach	Reach a decision with your patient	“Given your preference to avoid unnecessary medication and the likelihood that this could be viral, I suggest we hold off on antibiotics until the test results come back. Does that sound reasonable to you?”
Evaluate	Evaluate your patient’s decision	“Let’s follow up tomorrow morning once the test results are in. If it turns out to be bacterial, we’ll start the appropriate antibiotic right away. In the meantime, I’ll recommend supportive care to help manage your symptoms.”

Across many health care settings, SDM enhances patient knowledge, reduces decisional conflict and anxiety, improves satisfaction, and can positively impact health outcomes and care utilization—particularly among disadvantaged groups [2]. Additionally, this can be achieved without increasing health care costs or the average duration of a patient encounter, which is important for clinicians [2]. However, several factors impede its effective implementation, especially in urgent and emergency care settings. These include encounter time constraints, significant patient volume, patient expectations for specific interventions prior to the clinical encounter, lack of provider training in SDM, lingering professional attitudes of some providers rooted in a “paternalistic” approach to patient treatment, and—perhaps most importantly—insufficient diagnostic information [4].

The translation of diagnostics into treatment decisions is dependent in part upon SDM between health care providers and their patients. However, in clinical settings like urgent and emergency care, a suboptimal SDM process impedes quality treatment. The practice of evidence-based medicine depends upon the use of appropriate diagnostic tests to avoid unnecessary procedures and their associated risks, such as over testing or undue financial burden. This approach, combined with SDM, ensures that the chosen diagnostics are both medically appropriate and aligned with the patient’s values and preferences [5].

In acute care settings, effective communication about the purpose and outcomes of diagnostic testing has been shown to reduce patient anxiety and frustration, which improves outcomes [6]. Therefore, it is essential to use all resources that enhance a clinician’s ability to effectively communicate new diagnostic information that drives treatment decisions. Results from molecular testing, particularly nucleic acid amplification tests and reverse transcriptase-polymerase chain reaction (RT-PCR), are postulated to be information to empower a SDM conversation in the ambulatory care setting during an evaluation of an infectious disease complaint.

## RT-PCR and SDM

Historically, infectious disease diagnostics were rooted in traditional methods such as culture and serological testing. These modalities are labor-intensive, lacking in sensitivity, and too time-consuming for rapid clinical decision-making. The development of PCR technology in the 1980s promoted the dissemination of rapid, accurate, and highly sensitive diagnostic information. PCR tests are faster and often more sensitive and specific than traditional diagnostic methods, making them a valuable tool in clinical settings for rapid and precise pathogen identification [7]. Advancements in RT-PCR instrumentation have led to the next iteration of PCR technology, building on the foundation by allowing for the simultaneous detection and quantification of multiple genetic targets (potential pathogens) in a single run. This enhances diagnostic efficiency by allowing testing of multiple organisms in parallel, saving valuable time and resources—an advantage that is particularly beneficial in ambulatory acute care settings. Molecular testing is available as both a point of care and a “send-out” test. More recently, “next-day” PCR testing, which allows for the detection of multiple pathogens from a single clinical sample in under 24 hours, has gained visibility for its enhanced clinical utility [8,9].

When clinicians receive rapid diagnostic results, they are better positioned to explain the significance of these findings to their patients [10]. Effective communication of understandable diagnostic information (both before and after testing) is a critical component of SDM, as it helps patients grasp the basis for different treatment options and their associated risks and benefits. In fact, when patients are provided with clear, evidence-based information, they are more likely to participate actively in their care [10]. This not only reduces anxiety, but also builds a strong, trusting relationship between patients and providers—a cornerstone of effective SDM [10].

Recent studies have demonstrated that integrating multiplex RT-PCR testing into routine management of respiratory infections can reduce unnecessary antibiotic prescriptions, decrease health care utilization, and yield significant cost savings for patients and providers [9,11,12]. Reducing unnecessary antibiotic prescriptions is a public health priority, as antibiotic resistance is associated with nearly 5 million deaths per year worldwide [13]. Patients in intensive care units have elevated risk for acquiring antimicrobial resistant infections due to the increased risk of transmission, exposure to antibiotics, intensity of the treatment, and use of invasive devices [14].

The rapid return of PCR results allows for a more targeted therapeutic approach, providing clinicians with firm evidence to justify delaying empiric antibiotic use—a strategy that enhances patient understanding and supports antimicrobial stewardship by reducing overuse and resistance [9,12].

In urgent care settings, where patient expectations often impede appropriate prescribing practices, SDM has proven to be an effective counter strategy [15]. By fostering collaborative discussions that weigh the benefits and risks of antibiotic use based on fast, accurate diagnostic testing, SDM not only improves patient engagement—rising from 33% to 93%—but also leads to more responsible prescribing practices, with appropriate prescribing rates increasing from 20% to 95% [16]. Additionally, SDM combined with antimicrobial stewardship programs is an effective strategy to improve accuracy of antibiotic selection, treatment duration, and dosing for many common respiratory or other infections [17].

## Future Directions and Implications

To further integrate PCR testing into the SDM framework, supportive policy measures and targeted provider education are essential. Policy reforms to compel health systems and payers to prioritize support and investment in broad access to PCR testing, especially in underserved and resource-limited settings are needed as outlined in a policy brief from the Duke Margolis Center for Health Policy [18]. In particular, policy reforms are needed to (1) accelerate FDA approval pathways for multiplex PCR platforms for high-burden diseases; (2) expand insurance coverage for multiplex PCR testing and remove cost-share barriers (such as copays and deductibles); and (3) link reimbursement to testing accessibility and diagnostic accuracy, to reward providers who use PCR effectively to improve patient care. Ensuring equitable access to accurate and timely diagnostic information independent of a patient’s socioeconomic status or insurance coverage is critical to support informed SDM conversations. Achieving this will depend on thoughtful investment in health care infrastructure and evidence-based consideration of where such diagnostic tools can most effectively enhance outcomes. Moreover, continuous professional development programs that focus on SDM and advanced diagnostic communication for providers are crucial. As agencies such as the Centers for Disease Control and Prevention, the Agency for Healthcare Quality and Research, and the Urgent Care Association build and offer continuing education, clinical support tools, and training on antibiotic stewardship, PCR testing may be included as a tool with SDM as a modality for more judicious antibiotic use. Embedding PCR through SDM in such training initiatives can help overcome existing barriers and ensure that clinicians are well-equipped to translate rapid diagnostic data (from PCR findings, for example) into patient-centered language that promotes comprehension at various knowledge levels [4,10].

Despite the promising potential of incorporating multiplex PCR testing into SDM, further research is needed to fully elucidate the long-term impacts of this approach on clinical outcomes, health care utilization, and cost-effectiveness. Leading health agencies including the Infectious Diseases Society of America (IDSA) and the Presidential Advisory Council on Combating Antibiotic-Resistant Bacteria (PACCARB) have advocated for more clinical and economic outcomes data on diagnostic testing as a method to combat inappropriate antibiotic prescribing [19]. New research discussed here is encouraging, but there remains a dearth of information, which may admittedly delay access to testing. Future studies should aim to assess treatment outcomes, quality of life, and satisfaction levels of both providers and patients over more extended periods. Longitudinal research that tracks the integration of advanced diagnostics with SDM will provide valuable insights into the benefits and limitations of this approach. These studies are critical for guiding clinical practice, informing policy decisions, and ultimately ensuring that the full potential of advanced diagnostic technologies is appropriately realized in patient care across multiple clinical settings.

## Conclusions

The management of respiratory infections remains a significant challenge in modern health care, emphasizing the need for the integration of advanced diagnostic technologies with patient-centered care strategies. PCR testing stands at the forefront of diagnostic innovation, offering rapid, accurate, and comprehensive pathogen detection. Yet, the benefits of this technology are only fully realized when coupled with robust SDM. By facilitating clear, timely communication of diagnostic information, next-day PCR testing empowers clinicians and their patients to collaborate effectively on treatment planning. This drives improved clinical outcomes and more efficient health care delivery. As the process evolves, prioritizing policies and practices that foster innovation and effective patient engagement will be essential. Targeted focus on clinician training, equitable access to advanced diagnostics, and the evaluation of long-term outcomes are essential for integrating PCR testing into the SDM framework and thus yield tangible improvements in outcomes. Ultimately, this approach may transform the management of respiratory infections by promoting informed, evidence-based, and patient-centered decision-making, thereby enhancing the quality and efficiency of health care for all.

## Abbreviations

IDSA: Infectious Diseases Society of America
PACCARB: Presidential Advisory Council on Combating Antibiotic-Resistant Bacteria
RT-PCR: reverse transcriptase-polymerase chain reaction
SDM: shared decision-making
